# Quality of Life Following Permanent Neurological Damage after Subarachnoid Block

**DOI:** 10.1155/2011/906905

**Published:** 2011-09-25

**Authors:** Yvonne Dabota Buowari

**Affiliations:** ^1^Medical Women Association of Nigeria, Rivers State Branch, Flat 4, Lane 1, Road 1, Marine Base Housing Estate, P.M.B. 5115 Port Harcourt, Rivers State, Nigeria; ^2^No. 11 Ihediohanma Street, Mile 2 Diobu, P.M.B. 5115 Port Harcourt, Rivers State, Nigeria

## Abstract

*Introduction*. Caesarean section is the commonest operation carried out in females of the reproductive age group. Spinal anaesthesia is commonly used for caesarean section with its risk. Permanent paralysis of the lower limbs following subarachnoid block is a rare complication but can occur even in the best of hands. *Case Summary*. This is a 29-year-old final-year university student now 34 years old who had emergency caesarean section for cephalopelvic disproportion in 2005 under spinal anaesthesia in a low-resource setting in a developing country. She developed permanent neurological deficit thereafter. She had urinary and faecal incontinence for one year. She lost one academic session at her school because of long hospital stay and is now confined to a wheel chair. She celebrated her daughter's fifth birthday in October, 2010. Although there is ability in inability, she is now disabled. *Conclusion*. It is important for clinicians to recognise the complications of subarachnoid block promptly to avoid delay in treatment and severe neurological deficits.

## 1. Introduction

Caesarean section is one of the commonest operations preformed in women. The use of regional anaesthesia has dramatically increased over general anaesthesia for caesarean section. Although epidural, spinal, and combined spinal epidural techniques have been advocated, most straightforward caesarean sections are now performed with single-shot spinal anaesthesia which has been found to be faster, provides a superior block, and is cost effective [1]. For both parturient and anaesthesiologist, the most feared complication of regional anaesthesia is neurological deficit. Fortunately, neurological deficits are very rare. Peripheral nerve injuries may occur. Spinal anaesthesia is a type of regional anaesthesia in which an anaesthetic is injected into the spinal canal through a fine needle. The incidence of severe neurological deficits following spinal anaesthesia is low [2]. When they do occur, they are of great concern to both the patient and the practitioner. Despite the low incidence, some patients reject spinal anaesthesia, because they fear this complication.

The risks of paralysis are extremely low. The actual incidence of neurological dysfunction resulting from bleeding complications is estimated to be 1 in 150,000 for epidurals and 1 in 220,000 for spinal anaesthesia. There are several reasons for preferring spinal anaesthesia to general anaesthesia for caesarean section [3]. Spinal anaesthesia is increasingly used for caesarean section. It is safe and effective. The primary reason for choosing spinal anaesthesia is that it helps to avoid the complications associated with general anaesthesia. There is a small risk of complications associated with spinal anaesthesia including potential paralysis.

Professor August Bier performed the first surgical operation using spinal anaesthesia at the Royal Surgical Hospital of the University of Kiel, Germany, on August 16, 1898, heralding the advent of major regional anaesthesia using neuraxial blockade [4]. No surgical procedure is entirely risk-free. The risk of complications associated with spinal anaesthesia including potential paralysis. Regional anaesthesia techniques have several advantages over general anaesthesia including risk of failed intubations and aspiration of gastric contents, avoidance of depressant agents, and the ability of the mother to remain awake and enjoy the birth experience. In addition, blood loss is decreased under regional anaesthesia for caesarean section. 

Quality of life is defined as a multidimensional aspect covering all aspects of life. It is a person's sense of well-being that stems from satisfaction or dissatisfaction with the areas of life that are important to him or her [5]. Quality of life can also be referred to as a person's subjective well-being and life to include mental and physical health, material well-being, interpersonal relations within and outside the family, work, and other activities in the community, personal development and fulfilment, and active recreation [6]. Quality of life comprises four underlying domains: health/functioning, socioeconomic domain, psychological/spiritual domain, and family domain [7]. The term quality of life is used to evaluate the general well-being of an individual and social terms. The term is used in a wide range of contexts including the fields of international development, health care, and politics. The World Health Organization Quality of Life (WHOQOL) group defined quality of life as an individual's perception of their position in life in the context of the culture and value system in which they live and in relation to their goals, expectations, standards, and concerns [8]. this is a broad ranging concept affected in a complex way by a person's physicals health, psychological state, level of independence, social relations, and relationship to salient features of their environment [9]. It is a function of biophysical, environmental, and social conditions. We present the quality of life of a young woman who developed paralysis of the lower limbs after subarachnoid block for emergency caesarean section.

## 2. Case Presentation

A 29-year-old primigravida (now 34 years), an undergraduate at 40 weeks gestation, had emergency caesarean section for cephalopelvic disproportion under subarachnoid block in 2005 in a low-resource setting. There was no prior bleeding diastasis or neurological deficit. There was no intercurrent illness. Her weight was 80 kg and height 1.62 m. The spinal anaesthesia was administered with the patient in the sitting position and was technically difficult. A live female baby was delivered (who is now five years old). She was unable to move both lower limbs 24 hours after surgery and had a swelling on her back. There was loss of sensation in the both lower limbs with power 0. Power was normal in the upper limbs. She had urinary and faecal incontinence. Magnetic resonance imaging, computed tomography, and myelography was not available at the centre and the nearest tertiary health facility. A diagnosis of spinal haematoma following spinal anaesthesia was made. Patient was counselled for surgical evacuation of the hematoma, but she declined that she was sure of the outcome and scared. She was placed on conservative management. She spent thirteen weeks in hospital and was discharged home on request but to continue physiotherapy. She was unable to sit up at that time. Five years after, she is no longer incontinent of urine and faeces. She can now sit up but unable to walk. She is now confined to a wheel chair. There is full recovery of sensory function of the lower limbs but no motor function. There is loss of muscle bulk of the lower limbs.

She was a final-year university student when this incident occurred. She had to stay away from school for one year because of the long in-hospital stay. She is now a sales girl at her sister's dress shop.

No one in the family is happy about her situation, because she is the first female in her family to have a caesarean section as the other females in family all had spontaneous vaginal delivery. Since she comes from an island in Nigeria, she encounters some difficulty visiting her hometown, which is only accessible by air and water from the city where she resides.

## 3. Discussion

The use of regional anaesthesia for labour and caesarean delivery has significantly increased due to both patient demand and the reduced risk of maternal mortality and morbidity [10]. Although regional anaesthesia is relatively safe, serious complications can occur [11]. Haematoma within the vertebral canal after obstetric regional anaesthesia is extremely rare. The swelling at the patient's back after the surgery was probably a haematoma. Facilities for magnetic resonance imaging, computed topography, and myelography were not available at the centre and the health facilities in the vicinity at that time. Arranging for the transfer of the client to a facility that have such services proved abortive at the time of the incidence. 

Permanent nerve damage following central neuraxial blockade is a devastating but fortunately rare event [12]. Numerous mechanisms have been described that may result in major neurological complications. Due to underreporting and medicolegal implications, the true incidence of neurological injury associated with central neuraxial blockade is unknown. Numerous studies have reported on the incidence of complications associated with regional anaesthesia in general. Increasing litigations against medical doctors is one of the reasons for not openly discussing some reports that may have medicolegal implications.

The term “quality of life” is used to evaluate the general well-being of individuals and societies. It is used in a wide range of contexts including the fields of international development, health care, and politics. This patient she lost one academic year and is now confined to a wheel chair. Even with her situation, her family tries to improve her quality of life. So, she does not feel as an outcast on a wheel chair. 

Spinal anaesthesia carries the risk of bleeding. Compression of nervous tissue secondary to the formation of haematoma can cause neurological damage, which if not diagnosed and treated in a timely fashion can be permanent [13]. Even when performed with the proper technique, spinal anaesthesia carries the risk of bleeding. After regional anaesthesia, bleeding disorder are the greatest risk factors for the development of spinal haematoma although there are other known factors such as difficult or traumatic insertion of the needle. In this patient, there was difficulty in establishing the subarachnoid block although the client did not have a prior bleeding disorder. Spinal anaesthesia has advantages such as reduced blood loss and decreased incidence of deep venous thrombosis and pulmonary complications. As the mother's airway is not compromised, there is reduced risk of aspiration of gastric contents causing chemical pneumonitis (Mendelson's syndrome). Other advantages are for the mother remaining awake for the birth thereby initiating early bonding with the baby, avoiding the risks with general anaesthesia, and facilitating effective postoperative pain relief and early ambulation. 

The possibility of a haematoma is a source of concern for the anaesthesiologist, because it can cause permanent deficits. Computed tomography, magnetic resonance imaging, or myelography confirms the presence of a large mass compressing the spinal roots [13]. These radiographic imaging techniques were not available at the centre where the client has her caesarean section at the time of the incident.

Spinal anaesthesia is commonly used for caesarean section. Serious neurologic complications after spinal anaesthesia are rare but do occur. The complications of spinal anaesthesia may be categorised as spinal postdural puncture headache, cardiovascular, and neurological. The most common are postdural puncture headache and hypotension [2]. 

In most cases, symptoms of spinal subarachnoid haematoma can be recognised within 24 hours of regional anaesthesia [14, 15]. Spinal subarachnoid haematoma is extremely rare following obstetric regional anaesthesia [12]. It is, however, considerable should the clot compress the spinal cord or caudal equina [13]. Subsequently, motor and sensory deficits are observed with paresis or paralysis, urinary retention, and faecal incontinence, which resolved in this client after 1 year. Treatment of spinal subarachnoid haematoma is usually surgical when the patients present with neurological deficits [15]. In many cases, good neurological recovery has occurred in patients who have undergone surgical decompression within 8–12 hours [16]. The key to avoiding these problems is to have appropriate strategies in place to minimise the risk of complications and to investigate them urgently in a logical and systematic manner if they arise. This client had her surgery in a low-resource setting with minimal equipments and refused surgical evacuation of the probably spinal haematoma after she was counselled for it. She is one out of many to have such a complication.

## 4. Conclusion

Regional anaesthesia for caesarean section is increasing in popularity and gradually replacing general anaesthesia because of high incidence of deaths from failed intubation, hypoxia, and Mendelson's syndrome that are associated with general anaesthesia [17]. All major regional anaesthetic techniques have the potential to cause significant neurological damage, and although the incidence of permanent damage is small, there are few treatments for serious neurological damage, and some carry significant morbidity and limited potential for improvement. Perhaps no complication is more perplexing or distressing than persistent neurological deficits following apparently routine neuraxial block. The nerve roots or spinal cord may be injured. Postoperative peripheral neuropathies can be due to direct physical trauma to the nerve roots [16]. Although most resolve spontaneously, some are permanent [16]. Some studies have suggested that multiple attempts during a technically difficult block as a risk factor. A clinically significant haematoma can occur following spinal or epidural anaesthesia particularly in the presence of abnormal coagulation or bleeding disorder. The pathological insult to the spinal cord and nerves is due to a mass effect compressing neural tissue and causing direct pressure injury and ischaemia. The need for rapid diagnosis and intervention is paramount if permanent neurological sequalae are to be avoided. When haematoma is suspected, radiological imaging must be obtained immediately, and neurosurgical consultation should be requested. 

Serious and permanent neurological complications in the obstetric population are rare [18]. Most neurologic complications following childbirth are intrinsic obstetric palsies. Direct trauma to the spinal cord and spinal nerves may occur during neuraxial anaesthesia. Early intervention is the key to success in managing this potentially devastating complication. Prompt diagnosis and early surgical management are indicated. 

Perioperative nerve injuries have long been recognised as a potential complication of spinal anaesthesia [19]. Severe or disabling neurologic complications rarely occur.

## References

[1] Riley ET, Cohen SE, Macario A, Desai JB, Ratner EF (1995). Spinal versus epidural anaesthesia for caesarean section: a comparison of time efficiency, costs, charges, and complications. *Journal of Anaesthesia & Analgesia*.

[2] Hatim H (2002). Complications of spinal anesthesia. *Mount Sinai Journal of Medicine*.

[3] Casey WF (2000). Spinal anaesthesia—a practical guide. *Update in Anaesthesia*.

[4] Van ZA, Goeving M (2000). August bier 1861–1949. *Regional Anesthesia and Pain Medicine*.

[5] Jaiyesimi AO, Sofela EA, Rufai AA (2007). Health related quality of life and its determinants in Nigerian breast cancer patients. *African Journal of Medicine*.

[6] Niemi ML, Leaksonen R, Kolil U, Waltimo O (1985). Quality of life 4 years after stroke. *Stroke*.

[7] Ferrans CE, Powers MJ (1985). Quality of life index: development and psychometric properties. *Advances in Nursing Science*.

[8] Whoqol Group (1998). The WHO quality of life assessment (WHOQOL): development and general psychometric properties. *Social Science and Medicine*.

[9] Skevington SM, Lotfy M, O’ Connell KA (2004). The world health organization’ s WHOQOL-BREF quality of life assessment: psychometric properties and results of the international field trial. *Quality of Life Research*.

[10] Koyama S, Tomimatsu T, Kanagawa T (2010). Spinal subarachnoid haematoma following spinal anaesthesia in patient with hellp syndrome. *International Journal of Obstetric Anesthesia*.

[11] Loo CC, Dahlgren G, Irestedt L (2000). Neurological complications in obstetric regional anaesthesia. *International Journal of Obstetric Anesthesia*.

[12] Anthony RD (2008). Nerve damage following central neuraxial blockade is it avoidable. *Mexicana de Revista Anaestesiologia*.

[13] Mendes FF, Brescianini BC, Schneider FG, Segabinazzi D (2007). Conservative treatment of haematoma after spinal anaesthesia: case report and literature review. *Revista Brasileira de Anestesiologia*.

[14] Domenicucci M, Ramieri A, Daolin S (2005). Spinal subarachnoid haematoma, our experience, and literature review. *Acta Neurochirurgica*.

[15] Kreppel D, Antoniadis G, Seeling W (2003). Spinal hematoma: a literature survey with meta-analysis of 613 patients. *Neurosurgical Review*.

[16] Goyal A, Dua R, Singh D, Kumar S (1999). Spinal subarachnoid haematoma following lumbar puncture. *Neurology India*.

[17] Chestnut DH (1997). Anaesthesia and maternal mortality. *Anaesthesiology*.

[18] Wong CA (2010). Nerve injuries after neuraxial anaesthesia and their medicolegal implication. *Best Practice & Research Clinical Obstetrics & Gynaecology*.

[19] Terese MD, Horlocker T (1998). Neurologic complications of spinal anesthesia. *Techniques in Regional Anesthesia and Pain Management*.

